# Role of the IL-33/ST2 Activation Pathway in the Development of the Hepatic Fibrosis Induced by *Schistosoma mansoni* Granulomas in Mice

**DOI:** 10.3390/ijms241210237

**Published:** 2023-06-16

**Authors:** Laura Maggi, Genil Mororó Araújo Camelo, Izabella Chrystina Rocha, William Pereira Alves, João Marcelo Peixoto Moreira, Thiago Almeida Pereira, Wagner Luiz Tafuri, Élida Mara Leite Rabelo, Ary Correa, Roselene Ecco, Deborah Aparecida Negrão-Corrêa

**Affiliations:** 1 Laboratório de Esquistossomose e Imunohelmintologia, Departamento de Parasitologia, Instituto de Ciências Biológicas, Universidade Federal de Minas Gerais, Belo Horizonte 31270-901, MG, Brazil; laauramaggi@gmail.com (L.M.); genilmororo@gmail.com (G.M.A.C.); izabella.bebel@hotmail.com (I.C.R.); peixotomoreira@gmail.com (J.M.P.M.); 2 Curso de Enfermagem, Instituto de Ciências Biológicas e Saúde, Universidade Federal de Mato Grosso, Barra do Garça 78698-000, MG, Brazil; 3 Laboratório de Parasitologia Molecular, Departamento de Parasitologia, Instituto de Ciências Biológicas, Universidade Federal de Minas Gerais, Belo Horizonte 31270-901, MG, Brazil; willip.alves@gmail.com (W.P.A.); rabelo@icb.ufmg.br (É.M.L.R.); 4 Institute for Stem Cell Biology and Regenerative Medicine, Stanford University School of Medicine, Stanford, CA 94305, USA; dealmeida.thiago@gmail.com; 5 Laboratório de Patologia das Leishmanioses, Departamento de Patologia, Instituto de Ciências Biológicas, Universidade Federal de Minas Gerais, Belo Horizonte 31270-901, MG, Brazil; wlrasotafuri@gmail.com; 6 Laboratório de Micologia, Departamento de Microbiologia, Instituto de Ciências Biológicas, Universidade Federal de Minas Gerais, Belo Horizonte 31270-901, MG, Brazil; ary.correa.62@gmail.com; 7 Setor de Patologia, Escola Veterinária, Universidade Federal de Minas Gerais, Belo Horizonte 31270-901, MG, Brazil; eccoro.ufmg@gmail.com

**Keywords:** *Schistosoma mansoni* infection, liver fibrosis, granuloma, collagen deposition, reticular fibres, IL-33/ST2 activation pathway

## Abstract

*Schistosoma mansoni* eggs retained in host tissues induce innate cytokine release, contributing to the induction of Type-2 immune responses and granuloma formation, important to restrain cytotoxic antigens, but leading to fibrosis. Interleukin(IL)-33 participates in experimental models of inflammation and chemically induced fibrosis, but its role in *S. mansoni*-induced fibrosis is still unknown. To explore the role of the IL-33/suppressor of the tumorigenicity 2 (ST2) pathway, serum and liver cytokine levels, liver histopathology, and collagen deposition were comparatively evaluated in *S. mansoni*-infected wild-type (WT) and IL-33-receptor knockout (ST2^−/−^) BALB/c mice. Our data show similar egg counts and hydroxyproline in the livers of infected WT and ST2^−/−^ mice; however, the extracellular matrix in ST2^−/−^ granulomas was loose and disorganised. Pro-fibrotic cytokines, such as IL-13 and IL-17, and the tissue-repairing IL-22 were significantly lower in ST2^−/−^ mice, especially in chronic schistosomiasis. ST2^−/−^ mice also showed decreased α-smooth muscle actin (α-SMA) expression in granuloma cells, in addition to reduced *Col III* and *Col VI* mRNA levels and reticular fibres. Therefore, IL-33/ST2 signalling is essential for tissue repairing and myofibroblast activation during *S. mansoni* infection. Its disruption results in inappropriate granuloma organisation, partly due to the reduced type III and VI collagen and reticular fibre formation.

## 1. Introduction

Fibrotic diseases, such as liver cirrhosis, pulmonary fibrosis, chronic kidney disease, and cardiovascular disease, are responsible for approximately 45% of deaths in industrialised nations, showing that fibrosis is a major global health problem and should be better understood [1,2]. Fibrosis has been associated with excessive tissue deposition of the extracellular matrix (ECM) due to the proliferation and activation of fibroblasts and myofibroblasts that result in exacerbated growth of the injured tissue, forming hard scars that destroy the normal architecture of the affected organ [3,4,5].

A wide variety of stimuli from different etiological origins, such as autoimmune reactions, allergic responses, tissue damage, and persistent infections, including parasitic diseases, can trigger fibrotic processes [3,4,5]. One of the main causes of disease in schistosomiasis, caused by infections with *Schistosoma* spp., is fibrosis, and the liver is the main affected organ in this case [6,7,8,9].

It is estimated that more than 250 million individuals are infected by *Schistosoma* spp., and approximately 780 million people live in areas at risk of infection [10]. Among all the known species causing human schistosomiasis, *Schistosoma mansoni* is the most prevalent in the human population and is the only species transmitted in the Americas [11,12]. The morbidity of schistosomiasis is related to parasite load, time elapsed since infection, rate of reinfection, nutritional status, and comorbidities [13,14,15]. These different factors will determine the type and intensity of the host’s immune response elicited during infection, and a chronic, exacerbated type-2 response has been associated with the degree of fibrosis in this disease [13,15].

The initial immune response to *S. mansoni* infection is predominantly a type-1 response, induced by larval and adult worm antigens [16,17]. When oviposition begins, egg antigens are responsible for a shift towards a type-2 immune response, thereby triggering a heterogeneous extracellular matrix deposition around the parasite egg, which confines the cytotoxic effects of secreted antigens and restrains tissue damage. However, the type-2 response also leads to tissue fibrosis and portal hypertension, which is associated with the severity of chronic schistosomiasis [18,19,20,21,22,23]. In parallel, an immunoregulatory response is induced and becomes predominant around the 11th week onwards in most infected individuals [24,25,26]. The induction of an immunomodulatory response reduces T-helper (Th)2 inflammation, affecting granuloma size and formation, and controls morbidity [25,27,28].

As many parasite eggs are swept through the hepatic portal system and become trapped in the liver parenchyma, the induction and modulation of the immune response in this organ are essential for the outcome of schistosomiasis mansoni [29,30]. In this disease, resident liver cells play a key role in granuloma formation, tissue remodelling, and fibrosis. Activation of hepatic stellate cells (HSCs) and myofibroblast differentiation are necessary for collagen deposition and fibrosis in liver tissues, participating in granuloma formation during schistosomiasis [7,31]. HSC activation can be influenced by a multitude of signals from other resident and infiltrating cells, as well as autocrine and paracrine signalling from HSCs [7]. Profibrotic cytokines, including transforming growth factor (TGF)-β1, connective tissue growth factor (CTGF), IL-13, and IL-33, which are stimulated by *S. mansoni* egg antigens during granuloma formation, can also activate HSCs, triggering their differentiation into myofibroblasts, thereby contributing to *Schistosoma*-induced fibrosis [32].

Therefore, fibrosis is preceded by inflammation, and elements of the innate and adaptive immune responses are fundamental for the induction and regulation of fibrotic processes [2]. IL-33 is an alarmin released by resident liver cells, such as hepatocytes, endothelial cells, and HSCs, when *Schistosoma* eggs injure the host’s tissues [33,34]. The biological functions of IL-33 depend on the activation of its only known receptor, the suppressor of tumorigenicity 2 receptor (ST2), which is expressed in several liver-resident and -infiltrating cells [35,36,37]. IL33/ST2 binding on type-2 innate lymphoid cells (ILC2s) and eosinophils activates IL-13 production [36,38,39]. This cytokine, in turn, binds to the IL-13Rα2 receptor in HSCs and activates extracellular signal regulated kinases 1 and 2 (ERK1-ERK2), subsequently triggering activin receptor-like kinases (ALKs) to activate Smad 1 and Smad 2, resulting in CTGF expression in HSCs and inducing the expression of type I and III collagen, and matrix metalloproteinases [7,38,40]. Moreover, recombinant IL-33 was capable of activating HSCs in vitro, leading to the expression of IL-6, TGF-β, α-SMA, and collagen [32]. The IL33/ST2 activation pathway also stimulates the early production of the Th2 cytokines IL-13 and IL-5, which increase the expression of cell adhesion molecules (CAMs) in endothelial cells and leukocytes, thereby promoting infiltration and activation of CD4^+^ T cells, macrophages, and eosinophils, besides activating tissue-resident cells, such as fibroblasts [10,23,41,42,43,44].

The production of IL-33 has been associated with cellular recruitment, activation, and ECM deposition in several experimental models of fibrosis. Bleomycin-induced lung fibrosis has demonstrated that IL-33 promotes macrophage differentiation and activation towards an M2 phenotype, collaborating with fibrosis [45,46]. Liver fibrosis induced by thioacetamide and CCl_4_ administration or *Helicobacter hepaticus* infection also showed the essential role of the IL-33/ST2 signalling pathway in the pathological alterations [47]. However, the role of IL-33/ST2 signalling in fibrosis caused by schistosomiasis remains poorly understood.

In experimental infections with *S. japonicum*, exogenous administration of IL-33 leads to larger liver granulomas and a stronger Th2 response, whereas inhibition of the IL-33/ST2 pathway hinders fibrosis, collagen deposition, and differentiation of HSCs into myofibroblasts [36,43,48,49]. In *S. mansoni* infections, Vanella et al. [34] reported that the disruption of IL-33/ST2 signalling does not alter granuloma size or fibrosis and only hampers the expression of Th2 cytokines when blocked, along with IL-25 and thymic stromal lymphopoietin (TSLP) signalling. On the other hand, results from a study by Maggi et al. [50] indicate that ST2 knockout mice have impaired modulation of granulomas in the chronic phase of the schistosomiasis mansoni, leading to larger granulomas with an increased cellular infiltrate, resulting in higher mortality.

Therefore, the effects of the IL-33/ST2 pathway on hepatic fibrosis and HSC activation and differentiation during *S. mansoni* infection require further elucidation. In the current study, we provide data demonstrating that IL-33/ST2 signalling is an essential step for HSC activation during experimental schistosomiasis, and disruptions in this pathway result in inappropriate granuloma organisation with altered extracellular matrix composition.

## 2. Results

### 2.1. Systemic Immune Response

**Figure 1:**
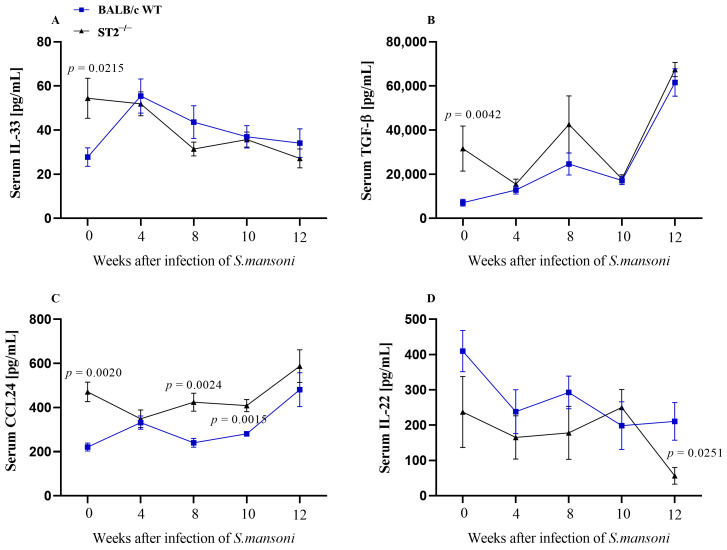
Serum cytokines levels. Evaluation of IL-33 (**A**), TGF-β (**B**), CCL24 (**C**), and IL-22 (**D**) levels estimated by enzyme-linked immunosorbent assay (ELISA) in serum samples from WT (blue squares) and ST2^−/−^ (black triangles) mice. Mice were subcutaneously infected with 50 *S. mansoni* cercariae and the serum was collected at weeks 4, 8, 10, and 12 post infection. Data are shown as kinetics and are presented as mean ± standard error of the mean (SEM). Normality was determined by the Kolmogorov–Smirnov test. Comparisons between normally distributed groups were carried out by Student’s *t*-test, and *p*-values were assigned. Differences between WT and ST2^−/−^ mice in the same phase of infection are represented by their *p*-value.

To assess the systemic immune response during *S. mansoni* infection, we quantified serum levels of IL-12p70, IL-13, IL-33, TGF-β, CCL24, and IL-22 (Figure 1). The majority of serum samples tested from both mouse strains had no detectable levels of IL-12p70 and IL-13. We also observed that uninfected ST2^−/−^ mice have higher serum levels of IL-33, CCL24, and TGF-β when compared with WT mice. When infected, IL-33 levels remain similar between mouse strains throughout the course of infection (Figure 1A). Moreover, as expected, the serum concentrations of the regulatory cytokine TGF-β increased progressively, peaking in the 12th week post infection in both WT and ST2^−/−^ mice (Figure 1B). In contrast, the serum level of CCL24, a chemokine that attracts eosinophils, and IL-22, a cytokine associated with tissue repair mechanisms, showed significantly different levels in WT and ST2^−/−^ infected mice. Serum levels of CCL24 were significantly higher in ST2^−/−^ mice compared with WT mice between the 8th and 10th weeks of *S. mansoni* infection; however, in the chronic phase of schistosomiasis, the serum levels of CCL24 were similar in both mouse strains (Figure 1C). IL-22 levels remained similar between the 2 strains until the 10th week of infection, while in the 12th week, the levels of this cytokine were significantly reduced in infected ST2^−/−^ mice (Figure 1D).

### 2.2. Liver Immune Response

**Figure 2:**
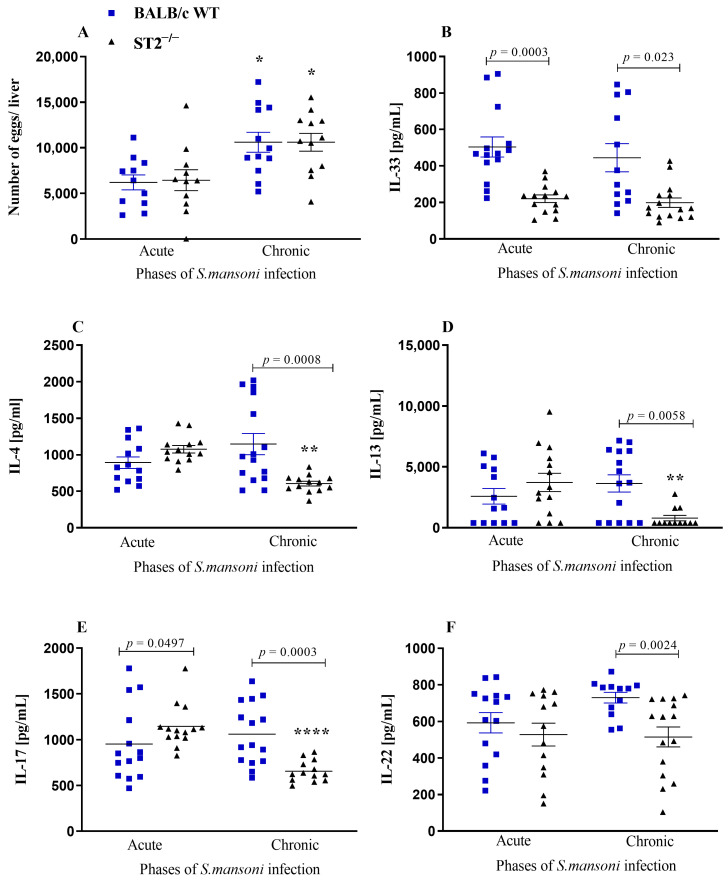
Parasite load and immunological parameters in the liver. Quantification of eggs retained in the liver of WT (blue squares) and ST2^−/−^ (black triangles) mice at 8 (acute) or 12 (chronic) weeks after infection with 50 cercariae (**A**). Quantification of Th2, Th17, and regulatory cytokines in liver homogenates from WT and ST2^−/−^ BALB/c mice during acute and chronic infections. Mice were subcutaneously infected with 50 *S. mansoni* cercariae and euthanised at weeks 8 (acute phase) or 12 post infection (chronic phase). Data compile results from two independent experiments and are presented as dot plots and mean ± SEM, each point representing an animal, of the IL-33 (**B**), IL-4 (**C**), IL-13 (**D**), IL-17 (**E**), and IL-22 (**F**) concentrations estimated by sandwich ELISA. Normality was determined by the Kolmogorov–Smirnov test. Comparisons between normally distributed groups were carried out by two-way ANOVA followed by Bonferroni’s post-test (**A**–**C**), while the Kruskal–Wallis test (**D**–**F**) was performed for non-parametric distributions, and the *p*-value were assigned. Differences between WT and ST2^−/−^ mice in the same phase of infection are represented by their *p*-value. * represents significant differences between chronically infected mice in comparison with mice of the same strain infected in the acute phase (* *p* < 0.05; ** *p* < 0.01; **** *p* < 0.001).

Consistent with our previous data [50], the number of *S. mansoni* eggs retained in the liver of both WT and ST2^−/−^ mouse strains was statistically similar throughout the infection (Figure 2A). Unlike that observed in serum, IL-33 levels in the liver are significantly lower in ST2^−/−^ mice compared with WT mice in both stages of infection (Figure 2B). Furthermore, we observed that egg retention induces the activation of a Th2 response even in the absence of the ST2 receptor, as seen by the concentrations of IL-4 and IL-13 in the liver; however, this response is not maintained in the chronic phase of infection, when ST2^−/−^ mice showed significantly lower levels of these cytokines than the infected WT mice (Figure 2C,D).

The concentrations of IL-17 in the liver homogenate were significantly higher in knockout mice compared with WT mice in the acute phase of infection, but the levels of this cytokine decreased significantly in ST2^−/−^ mice compared with WT mice in the chronic phase (Figure 2E). Just as in serum, the levels of IL-22 in ST2^−/−^ mice are significantly lower compared with WT mice during chronic schistosomiasis (Figure 2F).

### 2.3. Histopathology

**Figure 3:**
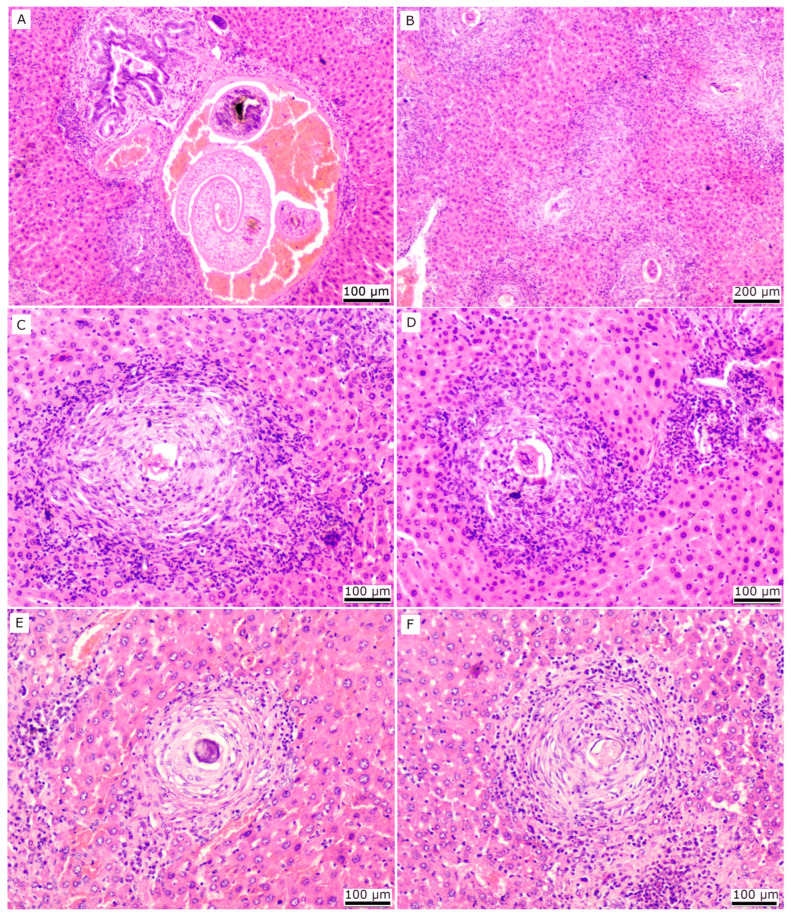
Histopathology of hepatic granulomas of WT and ST2^−/−^ mice infected with *S. mansoni*. Photomicrograph of a HE-stained liver section with sections of adult worms within the portal vessel of an infected ST2^−/−^ mouse (**A**). Photomicrograph of a HE-stained liver section slide of granulomas during acute phase from WT (**C**) and ST2^−/−^ (**B**,**D**) mice and chronic schistosomiasis from WT (**E**) and ST2^−/−^ (**F**). Scale bars = 100 µm (**A**,**C**–**F**) or 200 µm (**B**).

In order to analyse how the IL-33/ST2 pathway affects the formation of hepatic granulomas, we evaluated haematoxylin-eosin (HE) liver slides (Figure 3). Histopathological analysis revealed the presence of adult parasites in some liver sections within the portal vessels of both infected mouse strains. Transverse sections of the parasite revealed a thin tegument and digestive tube filled with a brown granular pigment (Figure 3A). Many vessels were hyperaemic and showed marked perivascular inflammation with moderate infiltration of plasma cells, lymphocytes, neutrophils, and eosinophils, associated with ductal proliferation (Figure 3A). In acute schistosomiasis, livers from both infected groups (Figure 3B) were characterised by multiple regions showing loss of parenchyma, which had approximately 40% of its area replaced by inflammatory cells associated with schistosome eggs and disorganised fibroblasts. Most of the inflammatory areas contained, centrally, a schistosome egg surrounded by numerous neutrophils, eosinophils, lymphocytes, and macrophages (Figure 3C,D). Fibroblasts intermixed with these inflammatory cells with minimal to moderate quantities of collagen fibres formed additional layers in the outer zone of these areas and were generally more intense in liver granulomas from WT mice (Figure 3C) than in ST2^−/−^ (Figure 3D). Infiltration of multinucleated giant cells was less frequent, and there were some pre-granulomatous areas. The remaining parenchyma among the well-circumscribed lesion areas had a preserved tissue architecture; however, scattered neutrophils and eosinophils were also seen between the cords of hepatocytes or some foci of lesion. These focal lesions were characterised by necrotic foci associated with fibrin, haemorrhage, moderate eosinophil infiltration, and macrophages containing cytoplasmic hemosiderin.

In chronic schistosomiasis, the liver sections of infected mice from the WT and ST2^−/−^ BALB/c groups were characterised by multifocal to coalescing areas with loss of parenchyma and replacement by well-formed granulomas associated or not with schistosome eggs. These areas replaced approximately 50–60% of the parenchyma. Inflammatory cell infiltrate was comprised of macrophages, multinucleated giant cells, lymphocytes, neutrophils, and eosinophils in different proportions in the different stages of the granulomas (Figure 3E,F). Some recent necrotic and acute inflammatory areas with viable eggs were scattered throughout the parenchyma. The periportal areas had numerous plasma cells, lymphocytes, and macrophages, and a mild increase in fibroplasia. Most of the liver granulomas from infected WT mice showed lower cellular infiltration and had organised layers of fibroblasts and eosinophilic fibres, concentrically arranged and surrounding the central area (Figure 3E), while liver granulomas from infected ST2^−/−^ mice still presented intense cellular infiltration and more disorganised fibre deposition (Figure 3F). In some granulomas, the eggs were disrupted and partially or completely mineralised, with less frequent eosinophils. Pigmented (hemosiderin-containing) macrophages were comparatively higher in these granulomas, although some were observed between the cords of hepatocytes.

In summary, the histopathological evaluation revealed differences in inflammation and fibre deposition in liver granulomas between infected WT and ST2^−/−^ BALB/c mice. Thus, the livers from these mouse strains were further evaluated to reveal the pattern of collagen deposition.

### 2.4. Hepatic Stellate Cell Differentiation

**Figure 4:**
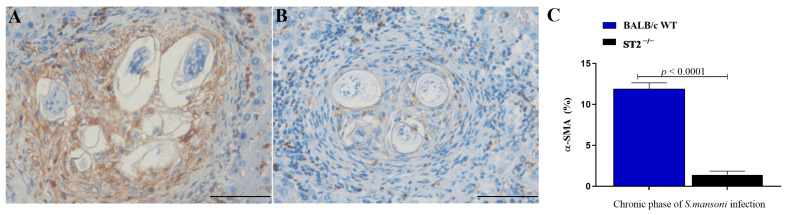
Analysis of myofibroblast activation in *S. mansoni*-induced hepatic granulomas. Photomicrographs of IHC-stained liver sections slides of granulomas from WT (**A**) and ST2^−/−^ (**B**) mice after incubation with anti-α-SMA antibody in chronic schistosomiasis. Scale bars = 50 µm. Morphometric analysis showing the percentage of the area marked with α-SMA per field in hepatic granulomas during the chronic phase of infection (**C**). The chart bars show the mean ± SEM of three mice per group. Normality was determined by the Kolmogorov–Smirnov test. Comparisons between the groups were carried out by the Mann–Whitney U test, and the *p*-value was assigned. Differences between WT and ST2^−/−^ mice in the same phase of infection are represented by their *p*-value.

As the differentiation of HSCs into myofibroblasts is characterised by the expression of α-SMA [7,51,52], the expression of this myofibroblast marker was evaluated in the livers of the experimental groups. Immunohistochemical (IHC) analysis revealed that many cells that composed *Schistosoma*-induced granulomas in the livers of infected WT mice expressed α-SMA, especially in the chronic phase (Figure 4A). In contrast, liver granulomas from infected ST2^−/−^ mice showed very few α-SMA-expressing cells (Figure 4B). Morphometric analysis of the brown-stained areas in hepatic granulomas of chronically infected mice from both mouse strains confirmed a significantly lower expression of α-SMA^+^ cells in granulomas formed in the absence of the activation of the IL-33/ST2 pathway (Figure 4C).

### 2.5. Collagen Deposition and Expression of Pro-Fibrotic Genes in Livers of S. mansoni-Infected Mice

**Figure 5:**
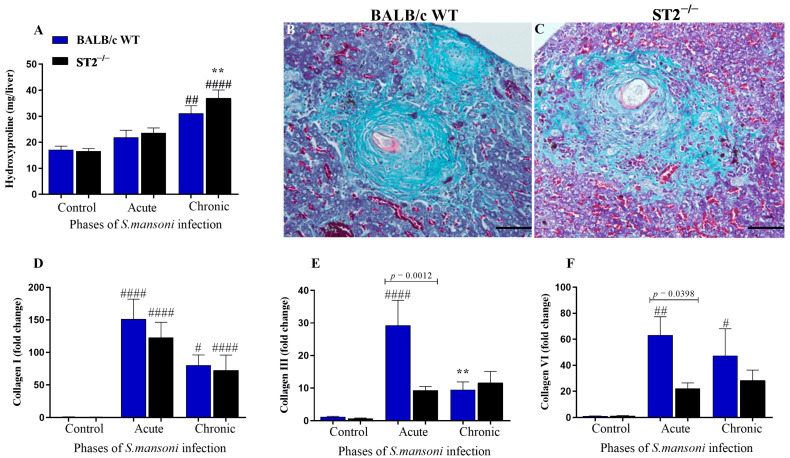
Evaluation of collagen induction and deposition in the liver of WT and ST2^−/−^ mice infected with *S. mansoni*. Quantification of hydroxyproline concentrations in the livers of WT and ST2^−/−^ mice after experimental infection with *S. mansoni* (**A**). Photomicrographs of Masson’s Trichrome-stained hepatic granulomas of WT (**B**) or ST2^−/−^ (**C**) mice chronically infected (14 weeks) with *S. mansoni*. Scale bars = 50 µm. Relative quantification of mRNA expression of *Col I* (**D**), *Col III* (**E**), and *Col VI* (**F**) in the livers of WT and ST2^−/−^ mice. RT-PCR was performed by extracting mRNA from the liver homogenate from uninfected WT and ST2^−/−^ mice or mice at weeks 8 (acute infection) and 12 (chronic infection) of *S. mansoni* infection. Chart bars show mean ± SEM of 12 mice per group (**A**) and 5 mice per group (**D**–**F**). Normality was determined by the Kolmogorov–Smirnov test. Comparisons between groups were performed by two-way ANOVA followed by Bonferroni’s post-test, and *p*-values were assigned. Differences between WT and ST2^−/−^ mice in the same phase of infection are represented by their *p*-value. # represents significant differences between infected mice and their uninfected controls; asterisk represents significant differences between chronically infected mice compared with mice of the same strain in the acute phase of infection (# *p* < 0.05; ** or ## *p* < 0.01; #### *p* < 0.0001).

To evaluate collagen production and deposition in the hepatic granulomas of WT and ST2^−/−^ mice, hydroxyproline levels were measured. The hydroxyproline concentration in the liver increased significantly during the chronic phase of infection in both WT and ST2^−/−^ mice; however, there was no difference between the strains (Figure 5A). Histopathological analysis of the liver parenchyma stained with Masson’s trichrome, a stain specific for collagen deposition, revealed that, although the hepatic hydroxyproline content in infected mice of both strains was similar, the pattern of collagen deposition in the granulomas showed marked differences between groups, especially in the chronic phase of *S. mansoni* infection (Figure 5B,C). In the chronic phase of schistosomiasis, the collagen deposition in the liver granulomas of infected WT mice was compact and organised in well-delimited fibres around the eggs (Figure 5B), while in infected ST2^−/−^ mice, the collagen was loosely and widely deposited around the parasite eggs with no well-defined fibre organisation (Figure 5C).

To assess the role of the activation of the IL-33/ST2 pathway on the expression of genes associated with liver fibrosis in infected ST2^−/−^ mice, qPCR analysis of collagen genes was performed (Figure 5). Regarding mRNA expression of *Col I* (Figure 5D), the *S. mansoni* infection induced a significant increase in *Col I* expression in WT and ST2^−/−^ mice, with no significant differences between mouse strains. As for *Col III* and *Col VI*, the infection induced a significant increase in the mRNA expression of both genes in WT mice, while in infected ST2^−/−^ mice, the expressions of *Col III* (Figure 5D) and *Col VI* (Figure 5E) were statistically similar to that of uninfected animals, both in the acute and chronic phases of schistosomiasis. Thus, the mRNA expression of *Col III* and *Col VI* in the livers of infected ST2^−/−^ mice was significantly lower than in infected WT mice (Figure 5E,F).

**Figure 6:**
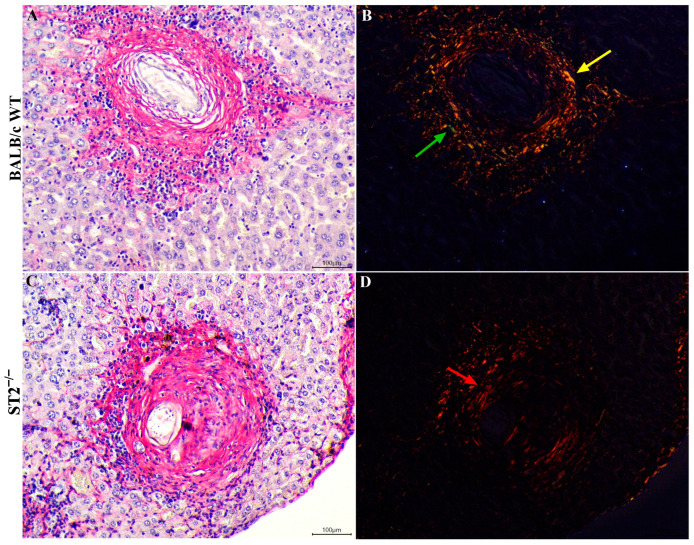
Collagen fibre deposition on Picrosirius Red staining in hepatic granulomas of WT and ST2^−/−^ mice chronically infected with *S. mansoni.* Photomicrographs of liver sections from infected WT and ST2^−/−^ mice stained with Picrosirius Red. Scale bar = 100 µm. Hepatic granuloma of WT mouse in chronic phase under optical microscopy (**A**) and under polarised light (**B**). Hepatic granuloma of ST2^−/−^ mouse in chronic phase under optical microscopy (**C**) and under polarised light (**D**). The green arrow indicates the presence of collagen III (greenish colour), and the yellow arrow indicates the presence of collagens I and III (yellowish colour) (**B**), while the presence of collagen I (reddish colour) is indicated by a red arrow (**D**).

The reduction of type III collagen fibres in liver granulomas of infected ST2^−/−^ mice was confirmed by Picrosirius staining. Although red-stained collagen fibres were observed in the granulomatous reactions around parasite eggs trapped in the livers of both mouse strains in chronic schistosomiasis (Figure 6A,C), the analysis of stained liver sections under polarised light confirmed that granulomas from WT mice (Figure 6B) presented red and green collagen fibres, which represent type I and III collagens, respectively, with overlapping type I and III fibres shown in yellow. In contrast, collagen fibres in liver granulomas from infected ST2^−/−^ mice were almost all red under polarised light, indicating a clear predominance of type I collagen (Figure 6D).

**Figure 7:**
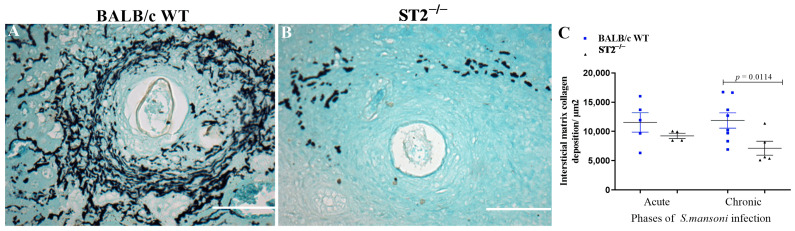
Reticular fibres of collagen in hepatic granulomas from *S. mansoni*-infected WT and ST2^−/−^ mice. Representative photomicrographs of liver sections from WT (**A**) and ST2^−/−^ (**B**) mice chronically infected with *S. mansoni* stained with Gomori’s Ammoniacal Silver. Scale bar = 50 µm. Morphometric analysis of reticular fibres in liver sections from WT (blue squares) and ST2^−/−^ (black triangles) mice during the acute and chronic phases of infection (**C**). Data are represented as a dot plot and mean ± SEM, each point representing the average of 20 granulomas per mice, with 4–8 mice per group. Normality was determined by the Kolmogorov–Smirnov test. Comparisons between groups were performed by Student’s *t*-test, and *p*-values were assigned. Differences between WT and ST2^−/−^ mice in the same phase of infection are represented by their *p*-value.

As indicated by Gomori’s ammoniacal silver staining of livers from *S. mansoni*-infected mice, the reduction of collagen III and VI deposition in hepatic granulomas formed in the absence of the activation of the IL-33/ST2 pathway was accompanied by modifications in the arrangement of fibres (Figure 7). As shown in Figure 7B and confirmed by morphometric analysis (Figure 7C), liver granulomas from chronically infected ST2^−/−^ mice showed a significant decrease in the formation of reticular fibres (Figure 7B,C). In contrast, WT mice developed granulomas with well-organised reticular fibres around the eggs, which can be seen as the black-stained regions on the periphery of the egg (Figure 7A).

## 3. Discussion

Parasitic diseases such as schistosomiasis are an important cause of liver fibrosis. Liver lesions caused by the *S. mansoni* infection are initiated by the retention of eggs in the hepatic capillaries, which activate elements of the innate and adaptive immune responses and lead to the formation of granulomas, a process that often results in liver fibrosis, associated with the severity of chronic schistosomiasis [7,24,53,54,55]. In the current study, we evaluated the role of the IL-33/ST2 activation pathway in the development of liver fibrosis caused by the experimental *S. mansoni* infection. Our data revealed a significant reduction of pro-fibrotic cytokines, such as IL-13 and IL-17, and the tissue-repairing IL-22 in the livers of chronically infected ST2^−/−^ mice. Moreover, infected ST2^−/−^ mice showed lower activation of HSCs and, consequently, lower differentiation of these cells into myofibroblasts. The decreased myofibroblast differentiation was accompanied by a reduced expression of type III and type VI collagen and lower formation of reticular collagen fibres in liver granulomas of infected ST2^−/−^ mice, indicating that the IL-33/ST2 activation pathway participates in liver-repairing mechanisms and the appropriate organisation of collagen around parasite eggs.

Interventions in particular immune pathways, such as IL-33/ST2 signalling, may impact pathology as long as they can interfere with broader immune-mediated mechanisms. Vannella et al. [34] have shown that the ablation of the IL-33/ST2 pathway during *S. mansoni*-induced inflammation may only significantly alter type-2 responses in the lung and liver in conjunction with the blockage of TSLP and IL-25. In addition, we observed in a previous work [50] that abrogation of the IL-33/ST2 pathway in experimental *S. mansoni* infections does not affect type-2 response, but is sufficient for inducing changes in global inflammation on its own, compromising immunoregulatory processes in chronic schistosomiasis.

Confirming our published results [50], the current data also demonstrated that, during experimental infection with *S. mansoni*, there is an activation of Th2 and Th17 responses, regardless of the absence of the IL-33 receptor; however, the IL-22 levels decreased systemically and locally. IL-22 is a cytokine homologous to IL-10, as their receptors share the IL-10Rb chain. Yet, unlike IL-10, which acts on immune cells, such as macrophages and T cells, IL-22 performs its function in tissue cells, such as hepatocytes [56,57]. During inflammatory processes, IL-22 directly protects the host tissue from destructive damage from the exacerbated immune response; in the liver, this cytokine promotes repair and tissue regeneration by stimulating the proliferation and survival of hepatocytes [57,58,59]. Aside from the possible role in tissue repairing, IL-22 and IL-10 may also play an important role in the modulation of liver fibrosis, decreasing HSC activation and tissue inflammation [59,60,61]. Interestingly, as demonstrated in our previous articles [50], ST2^−/−^ mice also showed a reduction in IL-10 levels from the acute to the chronic phase of the infection, which may also be associated with the exacerbated inflammatory response developed by this strain.

HSCs are resident cells in the hepatic parenchyma, and the transdifferentiation of these cells from their quiescent state to myofibroblasts results in pro-fibrotic and proliferative activity, which plays a central role in the pathogenesis of liver fibrosis induced by different harmful or inflammatory agents [62,63,64]. Experimental evidence indicates that activated HSCs are also a major source of collagen deposition and are essential for liver fibrosis and extracellular matrix remodelling in schistosomiasis [7,62]. However, the role of IL-33 in the activation of HSCs and liver fibrosis induced by the deposition of *Schistosoma* spp. eggs remain poorly understood [7,38,65]. Our data revealed that *S. mansoni* infection in ST2^−/−^ mice resulted in lower α-SMA expression in liver granuloma cells compared with infected WT mice. Since α-SMA expression is a marker of myofibroblast differentiation [7,51], this indicates that the IL-33/ST2 activation pathway is essential for the differentiation and activation of HSC-derived myofibroblasts in the livers of *S. mansoni*-infected mice.

The weak activation of HSCs in ST2^−/−^ mice, shown in this study, also corroborated the findings of Tan et al. [32] in an experimental model of bile duct ligation (BDL) fibrosis. HSCs isolated from ST2^−/−^ mice, even when stimulated with high concentrations (100 ng/mL) of recombinant IL-33, could not be activated, nor could they activate the c-Jun N-terminal kinase (JNK)/ERK/p38 transcription factors of the mitogen-activated protein kinase (MAPK) pathway, which are associated with the functionality of the IL-33/ST2 pathway, thereby indicating that the biological effect of IL-33 is dependent on the activation of the MAPK pathway and ST2 signalling in HSCs. By inhibiting the JNK/ERK/p38-MAPK pathway in HSCs isolated from C57BL/6 mice, a drastic decrease in collagen production was observed, indicating that IL-33/ST2 signalling through this pathway is essential for the activation and functionality of HSCs.

Furthermore, a previous study from our research group using the same experimental model showed that the concentration of IL-13, which is one of the major factors involved in the activation of HSCs and fibrosis, was also lower in the liver homogenate of ST2^−/−^ mice, while systemic and local TGF-β levels remain similar between both strains infected with *S. mansoni* [38,50]. Kaviratne et al. [66] demonstrated that IL-13^−/−^ mice infected with *S. mansoni* presented an almost complete abrogation of the fibrosis process, despite the continuous and unaltered production of TGF-β. The same study also demonstrated that, by inhibiting a part of the TGF-β signalling cascade during experimental schistosomiasis mansoni, there was no change in the development of fibrosis or the production of IL-13, suggesting that the process of liver fibrosis induced by *S. mansoni* infections, while dependent on IL-13, is TGF-β-independent. As demonstrated in other models of liver fibrosis, IL-13 can be activated via the IL-33 pathway, implying that the liver fibrosis mechanisms during schistosomiasis are mainly driven by a balance between IL-33 and IL-13 instead of TGF-β [7]. Our data further support that the sustained TGF-β levels are not sufficient for the induction of proper fibrosis in *S. mansoni* infections when the IL-33/ST2 pathway is not active. However, the lower levels of IL-13 during the chronic phase may have prevented appropriate extracellular matrix deposition, thus affecting granuloma formation in the absence of IL-33/ST2 signalling.

Other than that, a Th17 response has also been associated with granuloma formation and fibrosis [67,68], whose activation is related to TGF-β and IL-6 [69,70]. Our data show that the levels of IL-17 and IL-22, cytokines produced by Th17 cells, were affected by the lack of IL-33/ST2 signalling. The increased IL-17 concentrations during acute infection may have contributed to the observed unchecked periovular inflammation since this cytokine has been associated with increased granuloma size and pathogenesis in schistosomiasis [68,71]. Despite the lower IL-17 levels in chronic infection, they are accompanied by reduced IL-22 levels, both systemically and locally. The concomitant reduced IL-22 levels could have not been sufficient to compensate for the expectedly decreased IL-17-driven liver damage. Therefore, Th17-mediated pathogenesis could still be responsible for the maintained granuloma size during the chronic phase. Supporting our findings, Nady et al. [72] observed that the in vitro formation of granulomas induced by *S. mansoni* soluble egg antigen (SEA) significantly decreased in the presence of IL-22 and IL-17 together, while in the presence of IL-17 alone, there is granuloma growth due to the inflammatory role of this cytokine.

Despite the compromised differentiation of HSC-derived myofibroblasts during *S. mansoni* infection in ST2^−/−^ mice, the hydroxyproline content in the livers of these animals, an indirect measure of tissue collagen deposition, was similar to that of infected WT mice. Corroborating our data, Vannella et al. [34] also observed high and similar levels of hydroxyproline in the livers of IL-33^−/−^ mice infected with *S. mansoni*. Although our data showed that the tissue content of hydroxyproline was similar between the two strains of mice analysed, the differences in the deposition of collagen in the granulomas between WT and ST2^−/−^ animals, as portrayed by Masson’s trichrome staining, were markedly significant. ST2-deficient mice deposited collagen in a completely disorganised and exudative manner. Consistent with the increase in hydroxyproline in infected mice, there was an increase in *Col I* mRNA expression in the livers of infected WT and ST2^−/−^ BALB/c mice, whereas collagens III and VI increased only in the WT mice. Type I and III collagen production and deposition are commonly found in schistosomiasis granulomas, with collagen I being prominent during the chronic phase of schistosomiasis [6,73,74].

Our data showed that *Col I* expression was maintained in the absence of the IL-33/ST2 pathway, but the lack of the ST2 receptor significantly reduced *Col III* and *Col VI* expression in the acute phase of schistosomiasis, which was confirmed by histological analysis with Picrosirius staining under polarised light. The induction of *Col I* expression in infected ST2^−/−^ animals may be related to the initial increase in the liver levels of IL-17, as this cytokine is described as a potent inducer of type I collagen production [64,75,76]. Meanwhile, the reduction in type III collagen levels is in accordance with data found in the literature for the human and murine tendinopathy models, in which it was possible to relate IL-33 with type III collagen synthesis, showing that this cytokine plays a key role in the transition of type III collagen synthesis through the regulation of miRNA29a [77]. The lower production of type III and VI collagens in the absence of ST2 receptor activation resulted in a reduced deposition and greater disorganisation of reticular fibres in these animals. Reticular fibres are continuous collagen fibres composed of type I and III collagens in association with other collagens, such as V [78], and this network of fibres helps to protect the injured tissue, thereby contributing mainly to the containment of egg antigens [23,53]. As a result of this disorganisation in the deposition of the extracellular matrix and the significant reduction in the formation of reticular fibres in the *Schistosoma*-induced granulomas formed in ST2^−/−^ mice, these structures may not correctly confine the antigens released by *S. mansoni* eggs, developing greater microscopic liver lesions and, consequently, higher mortality [50].

This difference in the production of collagen types was observed in another experimental model, as demonstrated by Li et al. [45] in a model of pulmonary fibrosis induction in ST2^−/−^ and WT C57BL/6 mice subjected to inoculation with bleomycin. In these animals, although bleomycin stimulated type I collagen expression in both mouse strains, there was a significant reduction in *Col III* expression in ST2^−/−^ mice, which was associated with early fibrosis repair. When WT mice received mIL-33 together with bleomycin, an increase in inflammatory cells, such as alternatively activated macrophages (M2) and ILC2, was observed, which increased the production of IL-13 and TGF-β, soluble collagen, and the expression of *Col III*, consequently increasing the fibrosis score.

In addition to HSCs, alternatively activated macrophages play a special role in the fibrosis process. Alternatively activated macrophages typically express low levels of inflammatory cytokines and high levels of TGF-β, which promote collagen expression and fibrosis [79]. Experimental evidence indicates that M2 macrophages play a key role in the development of fibrosis and tissue repair in schistosomiasis [6,80,81]. Considering its role as an alarmin with a type-2 immune profile, IL-33 seems to be a potential activator of M2 macrophages during innate and adaptive immune responses, as they strongly amplify the expression of arginase I and cytokines that contribute to fibrotic processes [82]. Some authors have already reported this linkage of IL-33 with macrophages and the development of pulmonary [45], renal [83], and liver fibrosis due to *S. japonicum* infection [48]. However, there is still little clarification, and further studies are needed on the role of IL-33 in the activation and differentiation of macrophages involved in liver fibrosis during *S. mansoni* infection, which may be the key point for understanding the development of this process.

## 4. Methods

### 4.1. Mice

Six-to-eight-week-old female WT and ST2^−/−^ BALB/c mice were used in the experiments. WT BALB/c mice were acquired from an established colony at the university mouse facility. ST2^−/−^ mice, deficient in the receptor that is activated by IL-33 binding [84], were originally purchased from Jackson Immuno Research Laboratories and kindly provided by Dr. João Santana (Faculdade de Medicina de Ribeirão Preto, USP, Brazil). Both strains were maintained at the animal facility for infected animals of the Department of Parasitology (ICB, UFMG, Brazil). Experimental mice were fed standard chow (Presence, Primor, Brazil) and provided tap water ad libitum. The experimental procedures were approved by the Ethics Committee on Animal Use (protocols 104/2007, 159/2012, and 368/2018) of the Universidade Federal de Minas Gerais (UFMG, Belo Horizonte, Brazil).

### 4.2. Parasite and Infection

*S. mansoni* from the LE strain, originally isolated from a human patient in Belo Horizonte, Brazil, has been maintained by successive passages through *Biomphalaria glabrata* snails and *Mesocricetus auratus* hamsters at the Laboratory of Schistosomiasis and Helminth Immunology of the Department of Parasitology (ICB, UFMG, Brazil), and it was used for the experimental infections performed in this study. *S. mansoni* cercariae were harvested from infected *B. glabrata* snails, washed, counted, and subcutaneously injected into each mouse (25 or 50 cercariae per mouse, as defined for each experiment), as previously described by Pellegrino and Macedo [85]. To confirm parasite infection and burden, the left liver lobe of each infected mouse was digested in a 5% KOH solution, and the number of recovered parasite eggs was estimated, as described previously [86].

### 4.3. Experimental Design

To evaluate the role of IL-33/ST2 activation on the liver pathology and fibrosis associated with S. *mansoni* infection, WT and ST2^−/−^ BALB/c mice were infected with 25 or 50 cercariae and followed for up to 14 weeks. During this period, animals showing severe clinical signs of schistosomiasis, such as accentuated weight loss and apathy, were euthanised. At 0, 4, 8, 10, and 12 weeks after *S. mansoni* infection, 6 mice from each experimental group were immobilised, and blood was collected from the tail of each individual to quantify cytokines and chemokines. At weeks 8 and 12 or 8 and 14 post infection (acute and chronic phases of schistosomiasis, respectively), 6–10 mice from each experimental group (WT and ST2^−/−^) were euthanised by exsanguination via the brachial plexus under deep anaesthesia by intraperitoneal injection of ketamine (80 mg/kg—Dopalen—Sespo Industry and Commerce Ltda., Jacareí, Brazil) and xylazine (10 mg/kg—Kensol—Köing Laboratories S/A, Avellaneda, Argentine). The left liver lobes of mice infected with 50 cercariae were immediately removed and processed to quantify the gene expression of collagen types I, III, and VI using real-time quantitative polymerase chain reaction (qPCR). Pathological evaluation was carried out in experiments with 25 cercariae, and the chronic phase was analysed at 14 weeks. Due to the higher mortality rate of mice infected with a 50 cercariae inoculum, they were analysed at the early chronic phase, i.e., 12 weeks post infection. The left liver lobe was processed to estimate the number of parasite eggs retained in the parenchyma, the median liver lobe was harvested to estimate the hydroxyproline content, and the right liver lobe was promptly fixed and processed for histopathological analysis. Liver sections were subjected to immunohistochemical procedures or were stained using either haematoxylin-eosin, Masson’s trichrome, Picrosirius red, or Gomori’s ammoniacal silver.

### 4.4. Cytokine and Chemokine Assay

The concentrations of cytokines and chemokines were quantified in serum and liver homogenate of WT and ST2^−/−^ BALB/c mice by ELISA using commercially available kits (Duoset, R&D Systems, Minneapolis, MN, USA), following the manufacturer’s instructions. In serum samples collected at weeks 0, 4, 8, 10, and 12 post infection, the concentrations of IL-13, IL-12p70, IL-33, TGF-β, CCL24, and IL-22 were measured. To measure TGF-β, each serum sample was activated with a HCl solution and diluted, as described by the manufacturer. IL-4, IL-13, IL-33, IL-17, and IL-22 cytokine levels were quantified in liver homogenate samples of all experimental groups, as previously described [50].

### 4.5. Quantification of Hydroxyproline

As an indirect determination of collagen deposition, the hydroxyproline content was measured, as described previously [87]. A sample of the median liver lobe from each experimental mouse was homogenised in 0.9% saline solution, frozen at −80 °C, and lyophilised (Liotop K105—Liobras, São Carlos, Brazil). The assay was carried out with 20 mg of the lyophilised product that was subjected to alkaline hydrolysis and treated with chloramine T oxidising reagent, and the colorimetric reaction was assessed by the addition of Ehrlich’s reagent [88]. The standard curve for the assay was prepared using serial dilutions of known concentrations of hydroxyproline, and the absorbance was measured at 550 nm using a microplate reader (VersaMax, Molecular Devices, San Jose, CA, USA).

### 4.6. Histopathological, Immunohistochemical, and Morphometric Analyses of Liver

Histopathological evaluation of hepatic granuloma and collagen deposition was performed in liver sections from infected mice. Liver samples from each mouse were collected, fixed in 10% buffered formalin, embedded in paraffin, and cut into 5 μm thick sections. The liver sections were stained with haematoxylin-eosin to evaluate the overall inflammation and tissue damage. Masson’s trichrome and Picrosirius red staining were performed to illustrate extracellular collagen deposition around parasite eggs, and Gomori’s ammoniacal silver staining to characterise the reticular fibre organisation [89,90,91,92]. Moreover, picrosirius-stained slides were examined under polarised light microscopy (Opticam, O500R, São Paulo, Brazil) to characterise collagen composition; type I collagen appears as red fibres, and type III collagen appears as green fibres under polarised light [89]. The HE and picrosirius-stained images were captured with an Opticam LOPT14003 camera and analysed using OPTHD software (version x64, 4.10.17015.20200426). Liver sections stained with Gomori’s silver from infected mice in both the acute and chronic phases of schistosomiasis (6 mice/group) were used for morphometric analysis of reticular fibres. From each mouse, 20 microscopic fields containing granulomas were randomly selected, and the images were taken with an Olympus Bx40 microscope with an attached Qcolor 3 camera (Olympus, Tokyo, Japan). The area of the reticular fibres was quantified using the software package Ks-400 (version V.3.0) [93].

Immunohistochemistry (IHC) was performed on slides with 5 μm liver sections of mice from the different experimental groups to evaluate the expression of α-SMA. Each section was deparaffinised, rehydrated, and subjected to antigen retrieval using a 0.01 M sodium citrate solution (pH 6.0) by microwave heating at 80% power for 10 min. The slides were then incubated with anti-α-SMA antibody (Abcam 32575, Cambridge, MA, USA) at a dilution of 1:800 for 24 h at 4 °C. After incubation with the primary antibody, the slides were washed, and the presence of α-SMA in the liver parenchyma was revealed using the EnVision^+^ Dual Link System-horseradish peroxidase (HRP) kit (Agilent Dako, Santa Clara, CA, USA), in which the secondary antibody is associated with HRP-labelled polymers, and peroxidase activity was revealed by the chromogen 3,3′-diaminobenzidine (DAB; Agilent Dako, Santa Clara, CA, USA). All slides were counterstained with Harris modified haematoxylin (Sigma-Aldrich, St. Louis, MO, USA) for 2 min and with bluing reagent (Thermo Fisher Scientific, Waltham, MA, USA) for 1 min. Subsequently, the samples were analysed under an optical microscope, and α-SMA was quantitatively estimated in the livers of infected ST2^−/−^ and WT mice using the ImageJ software (version 1.43). The amount of α-SMA in granuloma cells was expressed as a percentage of the α-SMA-stained area per field, analysing exudative granulomas containing viable miracidia from chronically infected mice of both strains (3 mice/group), using an Olympus Bx41TF microscope coupled to a DP12-camera 2 (Olympus Optical, Tokyo, Japan).

### 4.7. Quantification of mRNA Expression by RT-qPCR

The quantification of gene expression of collagen types I, III, and VI was performed in liver samples using quantitative reverse transcription polymerase chain reaction (qPCR). Liver fragments of mice from different experimental groups were collected, stored in RNalater™ Stabilisation Solution (Invitrogen™, Waltham, MA, USA), and processed for RNA extraction using a tissue homogeniser (Omni TH) and TRIzol kit (Invitrogen), according to the manufacturer’s instructions. After the quantification of RNA in a spectrophotometer (Epoch™ Microplate, Biotek, VT, USA), the samples were subjected to complete digestion using a DNAse Turbo DNA-free kit (Ambion, Life Technologies, Thermo Fisher Scientific, Waltham, MA, USA), followed by cDNA synthesis using a commercially available High-Capacity cDNA reverse transcription kit (Ambion, Life Technologies). For quantification of gene expression, each PCR experiment was performed in triplicate in a total volume of 10 µL, combining 5 µL of SYBR Green Master Mix (Ambion, Life Technologies), 0.3 µL of each primer (forward and reverse) for the targeted gene, 2.4 µL of Milli-Q water, and 2.0 µL of cDNA. The specific primers for the collagen type I, collagen type III, and collagen type VI genes are listed in Table 1. The housekeeping gene GAPDH was used as an internal control. qPCR was performed in a StepOnePlus Real-Time PCR System (Applied Biosystems, Waltham, MA, USA) using the following parameters: Taq polymerase was activated at 95 °C for 10 min, followed by 40 cycles of amplification (95 °C for 15 s for denaturation, and 60 °C for 60 s for primer annealing and extension). Relative quantification of the mRNA expression for each target gene was performed using the 2^ΔCT^ method [94]. Data were analysed using StepOne 2.3 (Life Technologies).

### 4.8. Statistical Analysis

Statistical analysis was performed, and graphs were generated using GraphPad Prism 8.0.2. Data were subjected to ROUT tests for the identification and removal of outliers, and their normal distribution was verified using the Kolmogorov–Smirnov test. Comparisons between the experimental groups at different time points of infection were carried out by two-way ANOVA, followed by Bonferroni’s post-test, and comparisons between only one phase of infection were carried out by Student’s *t*-test. For non-parametric datasets, comparisons between multiple groups were performed by the Kruskal–Wallis test, while comparisons between only two groups were carried out by the Mann–Whitney U test. After being assigned, *p*-values ≤ 0.05 were considered significant.

## 5. Conclusions

The severity of schistosomiasis has been associated with the intensity and extent of tissue damage and fibrosis induced by parasite eggs trapped in the host’s tissues. This study shows that, in *S. mansoni*-infected mice, the IL-33/ST2 activation pathway did not significantly change the quantity of collagen deposited around the parasite eggs retained in the host’s liver. However, activation of this pathway participates in the induction of some pro-fibrotic cytokines, such as IL-13 and IL-17, and the tissue-repairing IL-22 in the livers of chronically infected ST2^−/−^ mice. Moreover, IL-33/ST2 signalling is essential for the differentiation of HSCs into myofibroblasts and, consequently, for the production of type III and VI collagen. This results in the proper organisation of collagen reticular fibres within the granulomatous reaction, enabling the adequate development of hepatic granulomas and antigen retention during schistosomiasis, protecting the liver parenchyma. Thus, the current study provides evidence that supports the role of the IL-33/ST2 pathway in liver-repairing mechanisms and the appropriate organisation of collagen around parasite eggs, participating in *S. mansoni*-induced liver fibrosis and controlling disease severity.

## Figures and Tables

**Table 1:** Primer sequences for GAPDH, Collagen type I, Collagen type III, and Collagen type VI used in the RT-qPCR.

Gene	Sequence (5′–3′)	
*GAPDH*	Forward	AGG TCG GTG TGA ACG GAT TTG
[95]	Reverse	TGT AGA CCA TGT AGT TGA GGT CA
*Col I*	Forward	ACT GGA CTG TCC CAA CCC C
[95]	Reverse	CTT AGT TTG GAC AGG ATC TGG
*Col III*	Forward	AAC CTG GTT TCT TCT CAC CCT TC
[94]	Reverse	ACT CAT AGG ACT GAC CAA GGT GG
*Col VI*	Forward	CGC CCT TCC CAC TGA CAA
[96]	Reverse	GCG TTC CCT TTA AGA CAG TTG AG

## Data Availability

All data supporting our results is present in the paper.
